# Universal Opt-Out Hepatitis C Virus Testing and Treatment on Entry in California State Prisons

**DOI:** 10.1001/jamainternmed.2026.4041

**Published:** 2026-09-14

**Authors:** Ziping Ye, Kimberley D. Lucas, Nathan W. Furukawa, Amanda A. Honeycutt, Donna Kalauokalani, Amy T. Krawiec, Teresa Puente, Joshua A. Salomon, Marissa B. Reitsma

**Affiliations:** 1 Department of Health Policy, School of Medicine, Stanford University, Stanford, California; 2 Medical Services, California Correctional Health Care Services, Elk Grove; 3 Division of Viral Hepatitis, National Center for HIV, Viral Hepatitis, STD, and TB Prevention, US Centers for Disease Control and Prevention, Atlanta, Georgia

## Abstract

This cross-sectional study investigates hepatitis C virus (HCV) testing and treatment outcomes among individuals entering California state prisons with universal opt-out HCV testing on entry.

Expanding testing and treatment of hepatitis C virus (HCV) infections is an essential component of national hepatitis C elimination plans.^1^ Compared with the general population in the US, state prison systems have substantially higher prevalence of current HCV infection (8.7% vs 1.6%), making them vital venues for testing and treatment.^3^ In July 2016, California Correctional Health Care Services (CCHCS) began implementing multiple strategies toward achieving elimination of hepatitis C within the state prison system, including universal opt-out testing on entry, gradual treatment eligibility expansions, and linkage to substance use disorder (SUD) treatment.^5^ With a universal opt-out approach, all entrants receive HCV testing regardless of their reported risk behaviors, unless they explicitly decline. This study characterizes HCV testing and treatment outcomes among individuals entering incarceration into California state prisons overall, by year, and by key individual-level characteristics.

## Methods

This cross-sectional study was determined by the Stanford University institutional review board to be non–human participants research because deidentified secondary information was used; therefore, written informed consent was not required. We followed the Strengthening the Reporting of Observational Studies in Epidemiology (STROBE) reporting guidelines. We analyzed individual-level electronic health record data from all adults entering California prisons, termed *entrants*, between July 1, 2016, and June 30, 2023. We quantified the percentages of entrants receiving an HCV antibody test within 4 weeks of entry, antibody positive (ever infected) among entrants tested, RNA positive (currently infected) among those with antibody positive results, and initiating direct-acting antiviral (DAA) treatment within 1 year among those with RNA positive results. To accommodate CCHCS care guidelines during the study period, which required a minimum sentence length of 5 to 8 months for DAA treatment, we restricted our analysis to entrants with at least 6 months of follow-up.

We reported on outcomes overall and by fiscal year (July 1 to June 30), and we stratified by sex, age group, documented race and ethnicity, prior incarceration in a California prison, anticipated length of incarceration, and SUD history. We identified predictors associated with testing, test positivity, and treatment using multivariable logistic regression. Additional methodologic details are provided in the eMethods in Supplement 1.

## Results

From July 2016 to June 2023, 176 967 individuals meeting eligibility criteria entered California prisons. Of entrants, 133 639 (76%) were tested for HCV antibody, 25 455 (19% of those tested; 14% of entrants) were ever infected with HCV, and 16 738 (66% of those ever infected; 9% of entrants) were currently infected (Table). A total of 7479 entrants (45% of those currently infected; 4% of entrants) initiated DAA treatment within 1 year. Among individuals initiating treatment within 1 year, 3837 (51%) started within 6 months of their positive test at entry. Among individuals who did not initiate DAA treatment, 4289 (46%) were released between 6 months and 1 year of entry. In a sensitivity analysis removing the 6-month follow-up restriction, the percentage initiating DAA treatment decreased from 7479 (45%) to 7613 (36%). From fiscal year 2017-2018 to 2022-2023, testing and treatment percentages increased (26 024 [80%] to 21 368 [92%] for testing and 675 [20%] to 1770 [76%] for treatment), antibody positivity remained largely stable (4628 [18%] to 4210 [20%]), and RNA positivity decreased (3327 [72%] to 2326 [55%]) (Figure).

**Table.** Hepatitis C Virus Testing and Treatment on Entry to California State Prisons by Demographic and Carceral Characteristics, Fiscal Years 2016-2017 to 2022-2023

Characteristic	Entrant, No.	No. (%)			
		Antibody test	Positive antibody test	Positive RNA test	DAA initiation
Overall	176 967	133 639 (75.5)	25 455 (19.0)	16 738 (65.8)	7479 (44.7)
Sex					
Female	10 631	10 041 (94.5)	1413 (14.1)	786 (55.6)	267 (34.0)
Male	166 336	123 598 (74.3)	24 042 (19.5)	15 952 (66.4)	7212 (45.2)
Age group, y^a^					
18-29	64 047	48 049 (75.0)	6096 (12.7)	4487 (73.6)	1951 (43.5)
30-49	91 852	69 858 (76.1)	14 270 (20.4)	9504 (66.6)	4359 (45.9)
≥50	21 068	15 732 (74.7)	5089 (32.3)	2747 (54.0)	1169 (42.6)
Race and ethnicity^b^					
American Indian or Alaska Native	1906	1531 (80.3)	472 (30.8)	263 (55.7)	110 (41.8)
Asian or Pacific Islander	2620	2027 (77.4)	97 (4.8)	52 (53.6)	23 (44.2)
Black	40 996	30 503 (74.4)	1760 (5.8)	1038 (59.0)	447 (43.1)
Hispanic	82 359	61 615 (74.8)	12 145 (19.7)	8487 (69.9)	3915 (46.1)
White	42 511	33 137 (77.9)	10 291 (31.1)	6422 (62.4)	2764 (43.0)
Other^c^	6575	4826 (73.4)	690 (14.3)	476 (69.0)	220 (46.2)
Previously incarcerated in a California state prison					
Yes	102 929	73 287 (71.2)	20 790 (28.4)	13 611 (65.5)	6207 (45.6)
No	74 038	60 352 (81.5)	4665 (7.7)	3127 (67.0)	1272 (40.7)
Time expected to serve, y^d^					
<1	60 809	48 288 (79.4)	9085 (18.8)	5876 (64.7)	1677 (28.5)
1-2	71 218	52 971 (74.4)	11 002 (20.8)	7222 (65.6)	3895 (53.9)
3-9	31 745	22 314 (70.3)	3894 (17.5)	2647 (68.0)	1421 (53.7)
≥10	13 195	10 066 (76.3)	1474 (14.6)	993 (67.4)	486 (48.9)
SUD status^e^					
Assessed, SUD identified	45 797	40 072 (87.5)	12 830 (32.0)	8365 (65.2)	4843 (57.9)
Screened negative or assessed, no SUD identified	60 739	50 259 (82.7)	5016 (10.0)	3108 (62.0)	1367 (44.0)
Not screened or assessed, no SUD program	70 431	43 308 (61.5)	7609 (17.6)	5265 (69.2)	1269 (24.1)
Fiscal year^f^					
2016-2017	31 328	4995 (15.9)	829 (16.6)	476 (57.4)	26 (5.5)
2017-2018	32 531	26 024 (80.0)	4628 (17.8)	3327 (71.9)	675 (20.3)
2018-2019	32 674	28 541 (87.4)	5483 (19.2)	3963 (72.3)	1716 (43.3)
2019-2020	20 845	18 568 (89.1)	3463 (18.7)	2426 (70.1)	752 (31.0)
2020-2021	13 467	12 165 (90.3)	2348 (19.3)	1460 (62.2)	845 (57.9)
2021-2022	22 788	21 978 (96.4)	4494 (20.4)	2760 (61.4)	1695 (61.4)
2022-2023	23 334	21 368 (91.6)	4210 (19.7)	2326 (55.2)	1770 (76.1)

Abbreviations: DAA, direct-acting antiviral; SUD, substance use disorder.

^a^ Age group is defined at entry.

^b^ Race and ethnicity groups are mutually exclusive. All races are non-Hispanic. Data collection for race and ethnicity is described in Supplement 1.

^c^ Individuals documented to be part of more than 1 race and ethnicity group were included in the other group.

^d^ Refers to the expected or observed sentence duration.

^e^ Routine SUD screening and assessment began in January 2020 (99.7% of individuals not screened or assessed entered before January 2020). Individuals were considered to have been identified with SUD if (1) their National Institute on Drug Abuse (NIDA)–Modified ASSIST Substance Involvement score indicated moderate risk or high risk (score of ≥4) for any substance category (cannabis, cocaine, prescription stimulants, methamphetamine, inhalants, sedatives, hallucinogens, street opioids, prescription opioids), (2) they had ever been treated with a medication for opioid use disorder, or (3) they met American Society of Addition Medicine Level of Care criteria for opioid treatment services. Individuals were considered to have no SUD identified if they screened negative on the NIDA Quick Screen or were not identified with SUD using any of the aforementioned criteria.

^f^ Fiscal year spanning July 1 to June 30.

**Figure.**
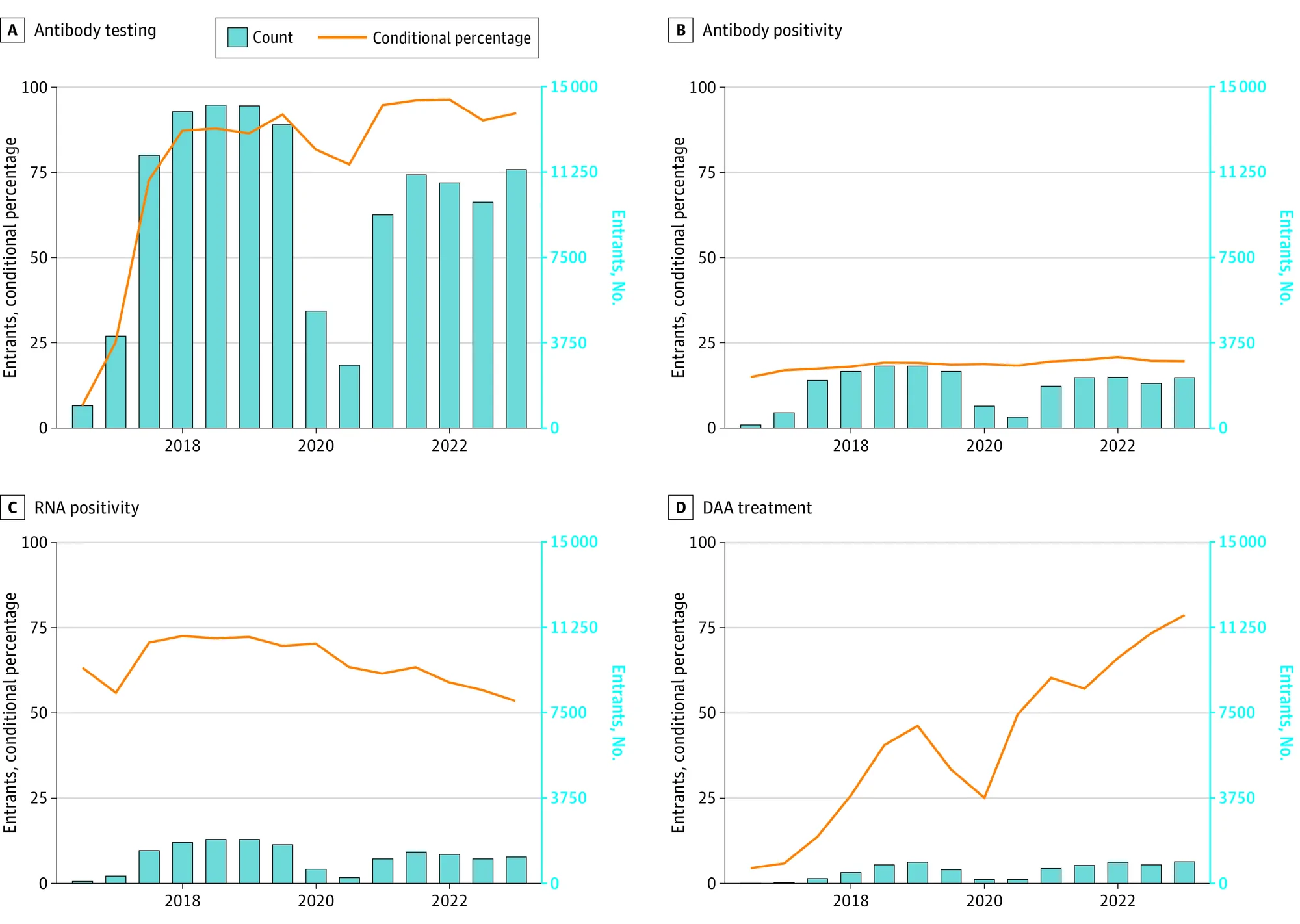
Bar and Line Graphs Showing Changes in Hepatitis C Virus Testing and Treatment on Entry to California State Prisons, Fiscal Years 2016-2017 to 2022-2023 Conditional percentages were calculated as follows: antibody testing is calculated among all entrants; antibody positivity is calculated among entrants with an antibody test; RNA positivity is calculated among all entrants with a positive antibody test; and DAA treatment is calculated among all entrants with a positive RNA test. The decline in antibody testing and DAA treatment observed in 2020 and 2021 coincide with the COVID-19 pandemic, which considerably impacted the prison health system. Lines connect values calculated over 6-month periods, and bars reflect counts over 6-month periods beginning with the period from July 1 to December 31, 2016. DAA indicates direct-acting antiviral.

Although antibody and RNA positivity percentages differed across age and racial and ethnic groups, treatment initiation was similar (Table). Current HCV infection prevalence among tested entrants was highest among male, older, and Hispanic entrants, as well as entrants who had previously been incarcerated in a California prison. Individuals with identified SUD had substantially higher antibody positivity (odds ratio [OR], 4.0; 95% CI, 3.9-4.2) and DAA initiation (OR, 1.4; 95% CI, 1.3-1.6), compared with individuals without an identified SUD.

## Discussion

CCHCS implemented universal opt-out HCV testing and treatment on entry as part of a wide-scale effort to eliminate hepatitis C; this implementation was associated with effective and equitable increases in access to hepatitis C treatment, particularly for those with SUD. Although we document subgroup heterogeneity in RNA positivity, the high overall current infection rate among entrants supports opt-out universal screening strategies.^3^ Furthermore, higher DAA initiation among individuals with current HCV infection and SUD underscores the value of integrated SUD and hepatitis C treatment programs. Our primary limitation is that results from California may not generalize to other states due to differences in prison systems, demographics, and epidemiology.

Efforts to expand testing and treatment were supported by changes to CCHCS’s HCV care-delivery model, including providing training and decision-support tools that enabled task shifting of clinical workups, treatment monitoring, and risk-reduction counseling to primary care teams and nurses.^5^ Additionally, treatment expansion required a commitment of up-front funding from the state of $105.8 million per year for 3 years.^7^ Although other studies have found that investments in treatment can yield downstream health care savings by preventing liver-related complications, future work should evaluate the long-term health and economic effects of CCHCS’s HCV testing and treatment strategies.^8^

## Notes

Supplement 1. **eMethods.** Eligibility and Data Processing Details

Supplement 2.
Data Sharing Statement

## References

[ild260020r1] US Department of Health and Human Services. Viral hepatitis national strategic plan: a roadmap to elimination for the US (2021-2025). Accessed July 28, 2026. https://www.hhs.gov/sites/default/files/Viral-Hepatitis-National-Strategic-Plan-2021-2025.pdf

[2] Akiyama MJ, Bialek T, Simonson R. The carceral-community cascade and HCV elimination. JAMA. 2025;333(5):369-370. doi:10.1001/jama.2024.20602 39541137 PMC12818903

[ild260020r3] Hall EW, Bradley H, Barker LK, . Estimating hepatitis C prevalence in the US, 2017-2020. Hepatology. 2025;81(2):625-636. doi:10.1097/HEP.0000000000000927 38739849 PMC11557732

[4] Spaulding AC, Kennedy SS, Osei J, . Estimates of hepatitis C seroprevalence and viremia in state prison populations in the US. J Infect Dis. 2023;228(suppl 3):s160-s167. doi:10.1093/infdis/jiad227 37703336 PMC11491819

[ild260020r5] Lucas KD, Krawiec A, Wada J, Kanan RJ. The hepatitis C care cascade in California state prisons: screening and treatment scale-up and progress toward elimination, 2016-2023. Clin Liver Dis (Hoboken). 2024;23(1):e0117. doi:10.1097/CLD.0000000000000117 38567093 PMC10986909

[6] McNamara M, Furukawa N, Cartwright EJ. Advancing hepatitis C elimination through opt-out universal screening and treatment in carceral settings, US. Emerg Infect Dis. 2024;30(13)(suppl 1):S80-S87. doi:10.3201/eid3013.230859 38561831 PMC10986823

[ild260020r7] State of California. 2018-2019 State budget 5225 Department of Corrections and Rehabilitation. Accessed July 28, 2026. https://ebudget.ca.gov/publication/e/2018-19/Department/5225

[ild260020r8] Chhatwal J, Aaron A, Zhong H, . Projected health benefits and health care savings from the US national hepatitis C elimination initiative. National Bureau of Economic Research. Published April 2023. Accessed July 28, 2026. https://www.nber.org/papers/w31139

[9] Congressional Budget Office. Budgetary effects of policies that would increase hepatitis C treatment. Accessed July 28, 2026. https://www.cbo.gov/publication/60407

[10] Abimbola TO, Symum H, Van Handel M, Teshale E, Thompson W. Lifetime medical costs of chronic hepatitis C in the US. BMC Health Serv Res. 2026;26(1):609. doi:10.1186/s12913-026-14360-1 41864952 PMC13126785

