# Supplementary material for: Universal Opt-Out Hepatitis C Virus Testing and Treatment on Entry in California State Prisons
Source: JAMA Intern Med. 2026 Sep 14:e264041. Online ahead of print. doi: 10.1001/jamainternmed.2026.4041 (PMC13575827; doi:10.1001/jamainternmed.2026.4041)
Supplement: Supplementary file 1 [file jamainternmed-e264041-s001.pdf]

Supplementary Online Content

Ye Z, Lucas KD, Furukawa NW, et al. Universal opt-out hepatitis C virus testing and treatment on entry in California state prisons. *JAMA Intern Med*. Published online September 14, 2026. doi:10.1001/jamainternmed.2026.4041

eMethods. Eligibility and Data Processing Details

This supplementary material has been provided by the authors to give readers additional information about their work.

© 2026 Ye Z et al. *JAMA Internal Medicine*.

eMethods. Eligibility and data processing details.

Eligibility

Our analytic cohort captured all adult individuals (ages 18 years and older) entering or re-entering the California state prison system at any point during July 1, 2016 through June 30, 2023 with at least six months of follow-up data. The six-month period was chosen to account for a one-month testing window, followed by a remaining sentence length of at least five months, which was the lower bound sentence duration required for DAA treatment eligibility during the study period. Depending on other clinical characteristics, treatment eligibility required a sentence length of up to eight months for some individuals. In 2024, the CCHCS's care guidelines were updated to remove the sentence length requirement for DAA treatment.

Data Processing

Demographic and Carceral Characteristics

We extracted data on demographic and carceral characteristics for individuals in our analytic cohorts. Demographic characteristics included documented sex (male, female), age group (18-29, 30-49, 50+ years), and race/ethnicity (Hispanic, non-Hispanic American Indian/Alaska Native, non-Hispanic Asian or Pacific Islander, non-Hispanic Black, non-Hispanic White, non-Hispanic Other). Carceral characteristics included whether an individual was previously incarcerated in the California state prison system (yes, no) and their sentence length (less than one year, one to two years, three to nine years, ten or more years).

Age was defined based on documented birth year at entry. Race/ethnicity groups are mutually exclusive. The Hispanic group included individuals of any race. Individuals with race/ethnicity documented as unknown or with multiple documented racial/ethnic groups were included in the non-Hispanic Other racial/ethnic group. Documented race/ethnicity is based on information previously recorded in an individual's official record of arrests and prosecutions (rap sheet). This field is usually self-reported, but in some cases may reflect classifications made by others.

Sentence length was defined as the time between entry and anticipated release. For current residents at the end of our data follow-up period, we used estimated release week to define sentence length. For current residents without an estimated release week, we assumed sentence length was 10+ years to reflect a lifetime sentence.

Hepatitis C Virus Testing

California Correctional Health Care Services hepatitis C virus (HCV) laboratory generally uses a single blood draw for HCV antibody and reflexed RNA tests. In some rare cases where prior HCV infection is documented, only RNA testing is performed. When constructing the HCV care cascade, we assumed a positive antibody test if an RNA test was performed without an antibody test. Since dates were aggregated to weekly indicators to protect privacy, if multiple tests performed in the same week yielded different results, we retained the positive test only.

Direct Acting Antiviral Treatment

We only included direct-acting antiviral (DAA) treatments for HCV in our analysis. As a result, a small number of individuals who were treated with pre-DAA regimens (e.g., pegylated interferon and ribavirin) were considered untreated in the present analysis. We used a one-year follow-up period from the date of entry to identify DAA initiation. The most commonly used DAA was sofosbuvir-velpatasvir (Epclusa) (90% of treatments), followed by elbasvir-grazoprevir (Zepatier) (7% of treatments), ledipasvir-sofosbuvir (Harvoni) (2% of treatments), glecaprevir-pibrentasvir (Mavyret) (1% of treatments), and sofosbuvir-velpatasvir-voxilaprevir (Vosevi) (<1% of treatments).

Substance Use Disorder (SUD) Assessment

Individuals were considered to have been identified with SUD if A) their NIDA-Modified ASSIST (https://nida.nih.gov/sites/default/files/pdf/nmassist.pdf) Substance Involvement Score indicated Moderate Risk or High Risk (score of 4+) for any substance category (cannabis, cocaine, prescription stimulants,

© 2026 Ye Z et al. *JAMA Internal Medicine*.

methamphetamine, inhalants, sedatives, hallucinogens, street opioids, prescription opioids), B) they had ever been treated with a medication for opioid use disorder, or C) they met American Society of Addiction Medicine Level of Care criteria (https://downloads.asam.org/sitefinity-production-blobs/docs/default-source/quality-science/asam_loc-assessment-guide-print_all.pdf) for opioid treatment services. Individuals were considered to have no SUD identified if they screened negative on the NIDA Quick Screen or were not identified with SUD using any of the aforementioned criteria. SUD screening and identification were defined using data from full incarceration periods (defined by entry and exit dates). Consistency within incarceration periods was implemented by applying the following hierarchy: identified with SUD, identified as not having SUD, not screened or assessed.

© 2026 Ye Z et al. *JAMA Internal Medicine*.
