# Supplementary material for: Universal Opt-Out Hepatitis C Virus Testing and Treatment on Entry in California State Prisons
Source: JAMA Intern Med. 2026 Sep 14:e264041. Online ahead of print. doi: 10.1001/jamainternmed.2026.4041 (PMC13575827; doi:10.1001/jamainternmed.2026.4041)
Supplement: Supplementary file 2 [file jamainternmed-e264041-s002.pdf]

Data Sharing Statement

Ye. Universal Opt-Out Hepatitis C Virus Testing and Treatment on Entry in California State Prisons. *JAMA Intern Med*. Published September 14, 2026.
doi:10.1001/jamainternmed.2026.4041

Data
Data available: No

Additional Information
Explanation for why data not available: Individual-level data from California Correctional Health Care Services are not publicly accessible and require a data use agreement. Analytic code and aggregate results are accessible at:
https://github.com/PPML/cchcs_hcv_screening_manuscript.
